# The Effects of Pecan Shell, Roselle Flower and Red Pepper on the Quality of Beef Patties during Chilled Storage

**DOI:** 10.3390/foods9111692

**Published:** 2020-11-19

**Authors:** Juliana Villasante, Manel Ouerfelli, Ares Bobet, Isidoro Metón, María Pilar Almajano

**Affiliations:** 1 Chemical Engineering Department, Universitat Politècnica de Catalunya, Av.Diagonal 647, 08028 Barcelona, Spain; julianavillasante@gmail.com (J.V.); manel.ouerfelli@upc.edu (M.O.); ares_16793@hotmail.com (A.B.); 2 Secció de Bioquímica i Biologia Molecular, Departament de Bioquímica i Fisiologia, Universitat de Barcelona, Joan XXII 27-31, 08028 Barcelona, Spain; imeton@ub.edu

**Keywords:** *Carya illinoinensis*, *Capsicum annuum*, *Hibiscus sabdariffa*, beef patties, meat lipid oxidation, antimicrobial activity

## Abstract

The antioxidant and antimicrobial effects of pecan shell (PSW), combined with roselle flower (RS) and red pepper (CA) were analyzed in beef patties by several methods during chilled storage for 13 days. Additionally, the antioxidant and antimicrobial activities of PSW, RS and CA extracts were determined. The PSW extract exhibited a higher radical scavenging activity (by the DPPH method) and more total phenolic compounds than RS and CA. RS presented the best antimicrobial capacity. Nine formulations of beef patties were prepared, including a control (CM), a synthetic preservative (CAMPA N.3 (A)) and different combinations of PSW, RS and CA. The bacterial counts of the beef patties with RS (4–5 log colony-forming units (CFU)/g meat) were significantly lower than those of the control sample (CM) (6–7 CFU/g meat) at day 6. The thiobarbituric acid-reactive substance (TBARS) values at day 7 of all treatments were similar to the values of samples containing the synthetic antioxidant and significantly lower than the CM group. The order of stability assessed by the TBARS values were in agreement with the hexanal content. Thus, these results support the hypothesis that the combination of PWS, RS and CA could represent a good natural food preservative.

## 1. Introduction

Beef for hamburgers contains a large amount of polyunsaturated and saturated fatty acids, proteins and minerals and shows a high water activity that makes the meat susceptible to oxidation and to microbiological damage. Additionally, bovine meat has a higher proportion of red fibers, containing iron and phospholipids, which are more sensitive to oxidation compared to white fibers. Additionally, minced meat experiences greater lipid oxidation than whole cuts, because the grinding process incorporates oxygen [1,2,3,4,5]. Therefore, as this process evolves, the sensory characteristics such as color, texture, flavor, odor, etc., and nutritional value of the beef meat, deteriorate [6]. Lipid oxidation produces many end products, including aldehydes, especially malondialdehyde (MDA). Meat color change, from pinkish-red to grayish-red, is unacceptable for consumers. This change is the result of the conversion of oxymyoglobin to metmyoglobin [2].

Synthetic antioxidants, such as butilate hidroxianisole (BHA) and butilate hidroxitoluene (BHT), are commonly used in the meat industry to delay oxidation and extend the shelf life of food. However, due to their potential risks to health and potential toxicity, there is a widespread agreement of the desirability of replacing synthetic antioxidants with natural antioxidants [7].

Several studies have focused on different by-products and aromatic plants as natural antioxidants, with the aim of protecting meat against oxidation. Ashrafuzzaman Zahid et al. have shown the application of clove extract in beef patties. This aromatic plant reduced protein and lipid oxidation, and also enhanced the quality of beef patties by increasing the redness value [8]. A by-product of olive leaves (extract) was effective against the oxidation of lipids and myoglobin during the storage of raw and cooked meat [9]. Ouerfelli et al. indicated that the incorporation of Azadirachta indica leaves in a beef patty formulation retarded the lipid oxidation and inhibited bacterial growth [10]. Furthermore, Villasante et al. studied the combination of pecans with roselle flower (RS) *(Hibiscus sabdariffa)* as an antioxidant and antimicrobial agent in sardine burgers [11]. They found that the combination of these plants is effective in extending the freshness and food safety of sardines.

This study is focused on three plants: pecans, red pepper and roselle.

The pecan *(Carya illinoinensis)* is a common dietary component in many countries around the world. Its shell is the by-product with the highest amount of polyphenols [12]. Previous studies reported that pecan shells contain bioactive compounds, including ellagic acid, gallic acid, chlorogenic acid, p-hydroxybenzoic acid, epigallocatechin, epicatechin-gallate and tannins [13,14,15]. Currently, there is no literature on this by-product in meat products.

Red pepper *(Capsicum annuum)* (CA) is commonly used to enrich food flavors. However, CA contains many phytochemical compounds, including flavonoids, phenols, carotenoids, capsaicinoids and vitamins, that make this type of pepper a good source of antioxidant, antimicrobial, antiviral, anti-inflammatory and even anticancer nutrients [16]. Paprika (*Capsicum annuum* L.) is a relevant spice, used in the production of meat products such as chorizo [17].

Roselle *(Hibiscus Sabdariffa)* (RS) flower is native to Asia and also widely cultivated in tropical and subtropical areas such as Central America and Africa [18]. This flower presents high contents of anthocyanins and flavonoids, including quercetin, delphinidin-3-sambubioside, delphinidin-3-glucoside, cyanidin-3-glucoside, cyanidin-3-sambubioside and kaempferol [19]. These compounds possess antioxidant and antimicrobial properties, and are also responsible for the prevention of chronic pathologies [20,21]. Several studies have worked with meat and this flower [22,23,24]. The purpose of this study is to determine the total phenolic compounds, anti-radical scavenging and antimicrobial activities of pecan shell, red pepper and roselle flower. For this reason, the effects of these plants on surface color, pH, lipid oxidation and microbial growth of raw beef patties during refrigerated storage at 4 ± 1 °C over 13 days were taken under study. Finally, a sensory analysis was conducted.

## 2. Materials and Methods

### 2.1. Plant Material

The nuts were collected in the north of Mexico (coordinates: 25°45′32″ N, 102°58′58″ W). The shells (PSW) were milled in a Wiley mill (Arthur Thomas, Philadelphia, PA, USA) equipped with a 2 mm screen. The red pepper (*Capsicum annuum*) (CA) was bought in a local market in Barcelona, Spain. The roselle flower (*Hibiscus sabdariffa*) (RS) was collected in Colima, Mexico (19°14′36′’ N, 103°43′30′’ W) and dried at room temperature at 25 °C for 1 month to obtain a final moisture of 12%.

### 2.2. Meat

Beef meat for the patties was collected seven days after slaughter, to allow it to mature. The meat was purchased from a local fresh food market in Barcelona, Spain, brought to the laboratory under refrigeration (4 ± 1 °C) and prepared in beef patties for testing during the same day.

### 2.3. Chemicals

All of the reagents were of analytical grade, Ethanol (EtOH), 2-thiobarbituric acid (TBARS), diphenyl-1-picrylhydrazyl (DPPH), Ethylenediaminetetraacetic acid (EDTA), Folin-Ciocalteu reagent, Ferric reducing antioxidant (FRAP), gallic acid (GA), 6-hydroxy-2.5.7.8-tetramethylchroman-2-carboxylic acid (Trolox), and penicillin; purchased at Sigma-Aldrich S.A. (Madrid, Spain). Methanol (MeOH), hydrogen chloride, Phosphate Buffered Saline (PBS), and ferrous chloride (FeCl2); acquired at Panreac Química S.L.U. (Barcelona, Spain). Miller Hinton agar and Tryptone glucose yeast agar were bought from Thermo-Fisher Scientific (Barcelona, Spain). Distilled water and Milli-Q water (Millipore, Barcelona, Spain). Synthetic antioxidant CAMPA N.3 (A) (dextrin, dextrose, 5.7% SO_2_, E-224, E-301, E-331) commonly used in the meat industry (Barcelona, Spain).

### 2.4. Extract Preparation

Extracts were prepared in order to determine the total phenolic content and to assay the radical scavenging activity (DPPH). Extraction was carried out using 1 g of PSW or CA in 10 mL of solvent (ethanol-water 50:50 *v*/*v*). One g of RS was weighed and extracted with 10 mL of 70:30 (*v*/*v*) ethanol-water. The PSW extract was stirred for 90 min at room temperature and RS extract at 60 °C. CA extract was kept under ultrasound for 30 min at 50 °C. All the extracts were centrifuged during 15 min at 16,800× *g*, and the ethanol-water supernatants were stored at −80 °C for 3 days.

### 2.5. DPPH Radical Scavenging Activity of the PSW, RS and CA Extracts

The assay was done according to the report of Gallego et al. [25]. Radical scavenging potential of PSW, CA and RS was evaluated using the DPPH method. The absorbance readings were measured by using a Fluostar Omega UV–Vis microplate reader spectrophotometer (Paris, France) at 517 nm, every 15 min for 75 min. The radical scavenging activity was expressed in μmol Trolox equivalents (TE)/g of dry weight (DW) ± standard deviation (SD). Measurements were done in triplicate for each sample.

### 2.6. Total Polyphenol Content (TPC) of the PSW, RS and CA Extracts

The TPC test was carried out according to the method described by Segovia et al. [26]. The total phenolic contents in the extracts were determined by the Folin–Ciocalteau method. The results were expressed as mg of gallic acid equivalents (GAE)/g (DW) ± (SD).

### 2.7. Antimicrobial Capacity of the Extracts of the PSW, RS and CA

Microorganisms were obtained from the collection of the Microbiology Department of the University of Barcelona, including Gram-positive *(Bacillus cereus* ATCC 11778, *Staphylococcus aureus* ATCC 25923, *Listeria monocytogenes* ATCC 15313, and *Micrococcus luteus* ATCC 4698) and Gram-negative (*Escherichia coli* ATCC 25922 and *Salmonella enterica* ATCC 14028) bacteria. Before using them experimentally, the microorganisms were cultured aerobically for 24 h at 37 °C in agar medium. The antimicrobial activity screening against Gram-positive and Gram-negative bacteria and the determination of the inhibitory effect of the different compounds were achieved by agar disc diffusion testing based on the technique described by Balouiri et al. [27]. Filter paper discs with a 6 mm diameter, each containing 80 µL of PSW, CA and RS extracts and ampicillin (100 µg/mL) as a positive control and Milli-Q water as a negative control, were placed on the agar surface. The Petri dishes were incubated at 35 °C for 24 h; eventually, the disc diameter of the inhibition zone around the extract was measured. The antimicrobial activity was determined in duplicate for each sample.

### 2.8. Formulations

#### 2.8.1. Preparation of the Beef Patties with PSW, CA and RS (Powder form on a *w*/*w* Basis)

Beef burgers were prepared (Table 1) in order to measure the evolution of oxidation by the thiobarbituric acid-reactive substances (TBARS) method, production of hexanal, % metmyoglobin, microbial quality and other physical characteristics, namely color and pH, throughout a period of 13 days under refrigeration at 4 ± 1 °C. Different sets of ground beef from different lots were used, each one taken from the round part of different cuts, and minced three times through 8 mm industrial plates at 4 ± 1 °C. The beef patties were prepared by simulating industrial practice. The water activity was measured using a water activity meter (AquaLab Pre Water Activity Meter, Pullman, WA, USA). The meat was mixed with sea salt (1.5% *w*/*w*) and assigned randomly to each treatment (Table 1). All batches (each 500 g of meat, for each treatment a total of 1500 g of meat were used) were mixed for 3 min to attain an even distribution of powders throughout the meat at a chilled temperature. Afterwards, each part was flattened and cut by a round cutter into small patties, and for each batch three patties were prepared; the patties used to determine fat oxidation weighed 8 ± 0.50 g, while the samples used for microbiological analysis weighed 10 ± 0.50 g. The patties were placed in plastic trays and covered with polyethylene film. The samples were stored in the dark at 4 ± 1 °C. All the chemical–physical analyses were performed on the same day. All the preparation was done aseptically. Triplicate analyses were performed for each sample. Three trials were carried out for the burger production.

#### 2.8.2. TBARS Assay

The antioxidant activity was evaluated based on the TBARS method described by Azman et al. and Grau et al. [28,29]. The absorbance of each sample was measured at 531 nm in a Fluostar Omega UV–Vis microplate reader spectrophotometer (Paris, France), and the results were expressed as mg malondialdehyde MDA/kilogram of meat. Eventually, 0.5 g of the sample were weighed and treated with 0.5 mL aqueous EDTA. Afterwards, using an Ultra-Turrax (IKA, Staufen, Germany) the sample was blended at 32,000 rpm for 1 min with 2.5 mL of thiobarbituric acid reagent. Then, it was filtered through a Whatman filter (no. 1) to obtain the solution. All procedures were performed while cooling in ice and carried out in the dark. The mixture was incubated at 95 ± 1 °C in water for 10 min. Then it was cooled for 10 min and subsequently the absorbance was measured. Samples were analyzed in triplicate in three different experiments on days 0, 3, 5, 7, 10 and 12. The percentage of inhibition was calculated using Equation (1):

(1) $$\%\;I=\frac{C-T}{C}$$

% *I*: Percentage of inhibition, *C*: value of TBARS (mg MDA/kg sample) of the control sample (CM) and *T*: value of TBARS (mg MDA/kg sample) of the sample.

#### 2.8.3. Determination of Hexanal by Headspace-Gas Chromatography Mass(HS-GC-MS)

The hexanal content was executed by HS-GC-MS, according to Villasante et al. [11], with the same conditions and materials. The raw meat (0.5 g) was mixed with Milli-Q water (1.5 mL) in a headspace vial. Afterwards, the vial was sealed to be airtight with a PTFE septum. The standard curve was prepared using hexanal (0.006 to 0.250 ppm). Results were expressed in mg hexanal/kg meat. The samples were analyzed on day 6.

#### 2.8.4. Microbiological Analysis

The meat samples (10 g) were diluted with 90 mL of Ringer solution and were placed in sterile 18 × 30 cm 400 mL Fisherbrand Stomacher bags (Fisher Scientific SL, Madrid, Spain) and homogenized for 1 min using a Seward Stomacher 400 (Seward Medical UAC House, London, United Kingdom). One hundred microliters of dilution were spread onto the surface of pour plates with agar (PCA). Subsequently, the plates were incubated at 35 ± 1 °C for 48 h. Afterwards, colony-forming units (CFUs) were counted and reported as log colony-forming units per gram (log CFU/g). The experiment was conducted in duplicate for each sample at day 0 and 6 of the study.

#### 2.8.5. pH Measurements

The pH of the samples was measured as a parameter of spoilage, since some hydroperoxide decomposition products are acidic. The pH value of six grams of beef patties was determined according Fan et al. [30] in triplicate using an electronic pH meter (Crison Instruments, GLP 21 pH METER, Barcelona, Spain). The experiment was performed on days 0, 6 and 13.

#### 2.8.6. Determination of Metmyoglobin (MetMb)

The metmyoglobin was evaluated according to the technique described by AMSA and Gallego et al. [31,32]. One gram of sample was mixed with 5 mL of 0.04 M phosphate buffer (pH 6.8), using an Ultra-Turrax (IKA, Staufen, Germany) for 30 s. The mixture was cooled 1 h at 4 °C. Then, at the same temperature, the sample was centrifuged at 4000× *g* for 10 min. The absorbance of the top phase was read at different absorbances (572, 565, 545 and 525 nm) using a Fluostar UV–Vis microplate reader spectrophotometer (Paris, France) on days 0, 3, 5, 7 and 10 of the experiment. The percentage of metmyoglobin was calculated using Equation (2):

(2) MetMb (%) = [1.395 ((A572−A700) / (A525−A700))] × 100

#### 2.8.7. Color Measurements

The color of the hamburgers was evaluated in triplicate at different points with an approximate angle of 10° by using a Minolta Chromameter CR-300. The patties were on a white surface at chilling temperature. The luminosity was determined by L*, red–green by a*, and yellow–blue by b*, with a bloom time of 30 min. The illuminant was D_65_. The chrome (c*) and hue angle (*h*°) were calculated with Formulas (3) and (4). The equipment was calibrated using a black and white glass tile. The color was evaluated on days 0, 6 and 13 of the experiment.

(3) $$c*\;=\;\sqrt{a^{*2}+b^{*2}}$$

(4) $$h{}^{\circ}\;=\;\tan^{-1}\;(b^{*2}/a^{*2})$$

#### 2.8.8. Water-Holding Capacity

The water-holding capacity (%) was determined according to the cooking loss (the amount of water that was lost when cooking the beef patties). The weight percentage difference between uncooked (*UNC*) and cooked samples (*C*) was calculated using Formula (4) [9]. The patties were stored in a freezer (−20 °C), then they were defrosted slowly in the fridge for 24 h at 4 °C. Beef patties were cooked at 80 °C for 5 min.

(5) Cooking loss %=((UNC(g) − C (g)) / UNC (g)) × 100

#### 2.8.9. Sensory Analysis

Sensory analysis was performed for five types of samples: CM (T1), 0.7% CAMPA N.3 (A) (T9), 2% PSW + 0.35% CA + 2% RS (T7) and 4% PSW + 0.35% CA + 2% RS (T8). Thirty-one untrained panelists (19 males and 12 females, aged between 18 and 60 years), students and workers from the University Polytechnic of Catalunya, evaluated beef patties (10 g) of each formulation studied. The patties were prepared the same day of the sensory evaluation according to AMSA (2015) [33]. A medium plate grill (George Foreman, Pectrum Brands INC, USA) was used, with a temperature of 200 °C. The patties were removed when they reached the desired internal temperature of 71 °C. The samples were presented to each panelist randomly on a white plate at 37 ± 1 °C, accompanied with an unsalted cookie and a glass of water to clean the palate. Each sample was coded with a randomly selected three-digit number. The panelists evaluated the following attributes: aroma, color, flavor, texture and overall acceptability, using a hedonic scale (9 = strongly like, 1 = strongly dislike). The purchase intention was also evaluated using a scale (9 = certainly would buy the product and 1 = certainly would not buy the product).

### 2.9. Statistical Analysis

Statistical analyses were conducted by using Minitab statistical software, version 18 (Minitab Inc., Sate College, PA, USA). The data were reported as means ± standard deviation. The results were subjected to analysis of variance (ANOVA), and then a post hoc Tukey’s test was applied to determine significant differences among formulations (*p* < 0.05).

## 3. Results and Discussion

### 3.1. Determination of DPPH and TPC of PSW, RS and CA Extracts

Several studies demonstrated that natural compounds with antioxidant properties are capable of inhibiting oxidation processes in food models and can provide health benefits [15,34]. Between the three samples studied, there were significant differences in the total amount of polyphenols (Table 2). The extract that contained more TPC was PSW, followed by RS and, finally, CA, with a low value. Similar results in Mexican PSW were obtained by [35] (92.5 ± 9.00 mg GAE/g DW). On the other hand, Kureck et al. [12] found in a shell aqueous extract (186.02 ± 2.31 mg AGE/g DW) almost two times more polyphenol than that reported by De la Rosa et al. [35]. The difference in the results could be due to the origin of the sample and to the method used to make the extracts. In previous studies, Villasante et al. [11] and Borrás-Linares et al. [36] reported similar values of the total amount of polyphenols in RS extracts, 22.40 ± 0.02 and 28.0 ± 4.00 mg GAE/g DW, respectively; in both cases, the RS samples used were from Colima, Mexico. CA was the sample containing the lowest TPC compared to the other plants. According to Hallmann et al., the TPC of different sweet bell pepper samples (*Capsicum annuum* L.), grown under organic and conventional growing systems, was between 953.6 ± 141.0 and 724.1 ± 115.5 mg GAE /kg DW [37]. These values are lower than the result reported in this study. Farhoudi et al. reported 3.38 ± 0.03 mg GAE/g DW for a jalapeño pepper [38].

The radical scavenging activity of PSW, RS, and CA extracts was determined (Table 2). The radical scavenging activity was significantly higher in PSW than in CA and RS. The result obtained for PSW was four times lower than that found in the Wichita variety (467.54 ± μmol TE/g DW) by Flores-Córdova et al. [39]. In the case of RS, it presented a similar antiradical scavenging activity to that reported by Villasante et al. [11] (78.66 ± μmol TE/g DW). The CA extract shows the lowest DPPH activity (73.06 ± 0.01 μmol TE/g DW).

### 3.2. Antimicrobial Capacity of PSW, RS and CA Extracts

The meat industry aims to control lipid oxidation, but also to develop safe products [40]. The antimicrobial activity of PSW, RS and CA was determined with the objective to use them as antimicrobial agents. The results show (Table 3) that RS was the sample that exhibited more antimicrobial activity in relation to all the microorganisms studied. In the case of *E. coli* (Gram negative) and *S. enterica* (Gram positive), penicillin showed less resistance than RS. On the other hand, PSW also showed antimicrobial capacity towards five out of the six microorganisms studied. Previous studies of CA determined a high antimicrobial activity [16,41] but in the present study, the CA extract showed poor activity; in fact, the CA studied showed less antimicrobial efficacy than PSW and RS, and only showed a moderate effect against *L. monocytogenes* and *M. luteus* microorganisms (Gram positive). This result confirmed the finding reported by Zhang et al. [42], who found that spice extracts are more active against Gram-positive than Gram-negative bacteria.

### 3.3. TBARS Assay (Meat Lipid Oxidation)

Meat products, when stored for a long time, suffer changes in their physical and chemical characteristics. These changes include the formation of free radicals, which are the cause of lipid oxidation [4]. Table 4 shows the evolution of the TBARS value (mg of malondialdehyde (MDA)/kilogram of meat sample) and, consequently, the degree of oxidation of the samples during the experiment. The CM group had a significant difference compared to the other samples. This fact demonstrated the antioxidant activity of CAMPA N. 3 (A), PSW, CA and RS and their synergies. After 7 days at 4 ± 1 °C, significant differences (*p* < 0.05) were already observed between samples. The T9 sample contained almost double the level of oxidation products assessed by mg of MDA per kg of meat than the T5 sample. The T6 sample had lower TBARS values compared to other samples at day 7. On the last day of the experiment, three groups of samples with similar degrees of oxidation were observed. The most effective treatments were the commercial preservative CAMPA N.3 (A) (T9), RS 2% (T5) and PSW 4% + CA 0.35% + RS 2% (T8), with a percent inhibition of 79, 73 and 75%, respectively, compared with the control (T1). Recently it was reported that lipid oxidation was significantly inhibited (*p* < 0.05) in pork patties treated with black currant extract, and TBARS values (% inhibition) decreased up to 91.7% compared with the control at day 9 of the experiment [43]. Additionally, in meat, Rodríguez-Carpena et al. investigated the effects of the waste (seed and peel) of two classes of avocado (Hass and Fuerte), and the percentage of inhibition ranged from 72.36 to 91.54 after 15 days [44,45]. So, the potential radical scavenging activity and the high TPC results exhibited by PSW, CA and RS extracts in vitro demonstrated that they could be used in a food model such as beef patties.

### 3.4. Determination of Hexanal by HS-GC-MS

Hexanal is a volatile secondary oxidation product that increases during the oxidation of meat and fish products. This by-product of lipid oxidation is generated mainly by the oxidation of polyunsaturated fatty acids [32]. At the beginning of the experiment, the amount of hexanal in the samples was below the detection limit (Table 5). At day 6, the concentration of hexanal in the CM group was significantly (*p* < 0.05) higher than in the other samples. However, the patties with T9 and T5 showed no hexanal formation. The other samples with PSW, CA and RS showed significantly (*p* < 0.05) lower quantities of hexanal compared to the control sample. There is a relation between the PSW percent increment in the sample and the amount of hexanal, but the values were not significantly different (*p* < 0.05) from those of the other samples. In a study reported by García-Lomillo et al. with seasoning derived from wine pomace in refrigerated beef patties, no hexanal formation was found, except for some low levels at the end of the experiment (day 15) [43]. They concluded that the seasonings were more effective than sulfites.

The TBARS assay is one of the most widely used methods for measuring secondary oxidation, but the determination of the hexanal content also supports the conclusions from MDA values presented in Table 4. The use of PSW, CA, RS and CAMPA N.3 (A) could be effective in preventing flavor and taste deterioration in beef patties.

### 3.5. Microbiological Analysis

The high water content and nutrients present in the beef meat made the patties extremely prone to bacterial growth. The water activity of the meat used was 97% and, according to the FDA, with this % of water activity, the pathogens grow easily [46].

**Figure 1:**
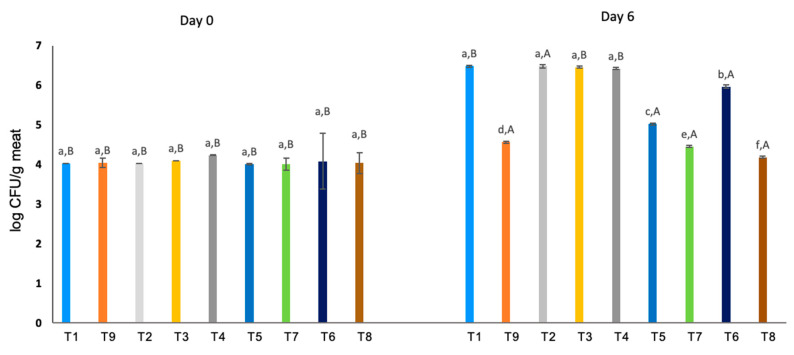
Microbiological analysis at days 0 and 6 in beef patties in refrigerated storage at 4 °C. Results are given with standard error. Different small letters, ^a–f^, across the same day indicate significant differences between samples and different capital letters, ^A,B^, in the same sample indicate significant differences between days. T1: CM (control), T2: 2% PSW, T3: 4% PSW, T4: 0.35% CA, T5: 2% RS, T6: 2% PSW + 0.35% CA, T7: 2% PSW + 0.35% CA + 2% RS, T8: 4% PSW + 0.35% CA + 2% RS and T9: 0.7% CAMPA N.3 (A).

Figure 1 shows the level of microbial contamination of the different samples on days 0 and 6 of the experiment. The results show that the samples which contained RS had a similar behavior to the patties with synthetic preservative (0.7% CAMPA N.3) after 6 days of storage, with values between 4 and 5 log CFU/g meat. These values were consistent with the results presented in the assessment of the antimicrobial activity.

However, for red pepper, the low antimicrobial activity of the extract is reflected in the results of Figure 1. The value of microbial contamination was similar to the CM group, between 6 and 7 log CFU/g meat, and did not have an inhibitory effect on microbial growth in meat.

The PSW also had moderate antimicrobial activity (Table 3). In the microbiological results (Figure 1), the T7 (2% PSW + 0.35% CA + 2% RS) and T8 (4% PSW + 0.35% CA + 2% RS) samples presented lower microbial growth than the T6 (2% PSW + 0.35% CA) and T5 (2% RS) samples. On the other hand, for the T2 (2% PSW) and T3 (4% PSW) samples, no decrease was observed in the microbial growth, and the values obtained were similar to the T1 (CM) sample. So, the results suggested that there is a synergy between PSW and RS in the prevention of microbiological contamination in raw meat. Similar results were reported in a study on the effect of RS and pecans *(Carya illinoinensis)* on antioxidant and antimicrobial activity in raw sardines [11].

### 3.6. pH

Table 6 shows the pH values of the samples at different days of the experiment. In the majority of the samples, the pH increased over time. Nevertheless, in the case of the treatments with 0.7% CAMPA N.3 (A) and all the samples with RS, the pH remained around 5 during the whole experiment (Table 6). The hibiscus flower showed a strong antimicrobial activity against different Gram-positive and Gram-negative bacteria. This could be directly related to the low pH values of the RS samples. On the other hand, according Pérez-Báez et al. and Da-Costa-Rocha et al., the incorporation of RS decreased the pH in the patties due the organic acids in this flower (pH = 2.3) [24,47].

A previous study investigating Agave angustifolia extract in pork patties, stored under an aerobic environment at refrigerator temperatures for several days, found an increase in pH (*p* < 0.05) [6]. These results could be due to the degradation of nitrogenous components in meat during the storage period. This process was directly influenced by the action of endogenous or microbial enzymes such as proteases or lipases [6,48].

### 3.7. Percentage of Metmyoglobin

**Figure 2:**
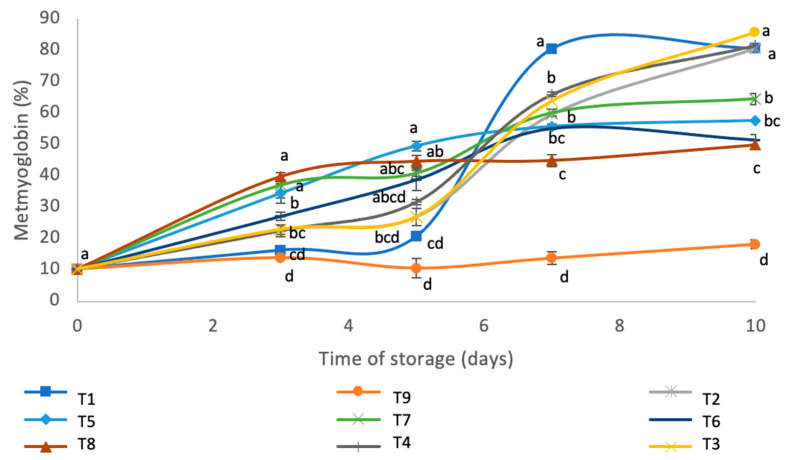
The % of metmyoglobin in beef patties during 12 days of refrigerated storage at 4 ± 1 °C. Different letters across the same day indicate significant differences between samples. T1: CM (control), T2: 2% PSW, T3: 4% PSW, T4: 0.35% CA, T5: 2% RS, T6: 2% PSW + 0.35% CA, T7: 2% PSW + 0.35% CA + 2% RS, T8: 4% PSW + 0.35% CA + 2% RS and T9: 0.7% CAMPA N.3 (A).

The evolution of the percentage of metmyoglobin in the samples, studied for 10 days’ storage in a refrigerator, is shown in Figure 2. Many factors could influence the color of the meat products, but the susceptibility to self-oxidation of myoglobin was the predominant factor. The change of color from red to brown during storage is associated with the oxidation of red oxymyoglobin (OxyMb) to brownish metmyoglobin (MetMb) [15,49]. The sample that exhibited least reduction in the percentage of metmyoglobin during the experiment was the commercial preservative (CAMPA No. (A)), which remained stable (*p* < 0.05) during the 10 days of storage at 4 ± 1 °C. However, the rest of the samples retained a high percentage of metmyoglobin for 10 days. The samples with least metmyoglobin, apart from CAMPA N.3 (A), were the T8 and T6, which had very similar values (49.85% and 51.35%, respectively). The samples with T5 and T7 had higher values of 57.61% and 64.49%, respectively. Finally, the samples with the highest percentage of metmyoglobin were: 2% PSW, CM, 0.35% CA and 4% PSW.

In general, the samples with RS contained a lower % of metmyoglobin than the rest and a stabilization of metmyoglobin formation was observed between days 7 and 10 of the experiment (no significant differences existed (*p* < 0.05)).

In agreement with the present results, the literature reported, after day 6 of storage, a stabilization of the % of metmyoglobin in beef patties treated with carnosine [50].

### 3.8. Color

The color of the raw beef, that could be affected by bacterial contamination and the formation of metmyoglobin, was dependent on low oxygen partial pressure, pH, water-holding capacity, lipid oxidation and other parameters such as diet, genetics and the breed of the animal [51,52]. The color values of the beef patties were characterized as L*, c* and *h*°* on every day of the experiment (Table 7). There was no significant difference in L* values between the control and the other groups. Similar results were reported by Araújo do Prado et al., who did not find any differences in L* values for beef burgers with added tannins [53]. However, the different treatments produced significant changes in values of both c* and *h*°, even in the same sample but on different days. The c* value of the control and the other samples decreased as storage time progressed, and higher c* values were associated with increases in the a* (red–green) and b* (yellow–blue) values. These findings were consistent with the results shown in Table 7, where a reduction in the color values a* and b* over time indicates a change of the meat color, from red (oxymyoglobin) to brown (metmyoglobin) [54].

Nevertheless, c* values over time decreased more in the samples containing natural additives than the CM or the CAMPA N.3 (A) samples, probably due to the composition of PSW, CA and RS. In the case of PSW, the high content of fiber affected the values, and these findings agreed with a literature report, which supports the hypothesis of gel formation between the fibers of the shell and the meat proteins, with a reduction of red coloration [55].

### 3.9. Water-Holding Capacity (WHC)

The water-holding capacity of the meat is an important quality parameter in the food industry, due to its economic implications [9]. Table 8 shows the results of the cooking loss in percentage of the samples: T1, T9, T6, T8 and T6. The less weight lost during the cooking process, the better the water-holding capacity.

All the samples had higher percentages than the control sample, but the cooking loss percentages between the samples did not vary significantly (*p* < 0.05). The sample that exhibited the highest cooking loss (%) contained 2% PSW + 0.35% CA + 2% RS (T7). The nature of the shell, which contained more than 58.90% fiber [39], could, in higher quantities, retain more water in the patties. Previous studies reported an increased WHC with the addition of dietary fiber into beef patties, probably due to the moisture and fat in the fiber matrix [56,57,58]. Gibis and Weiss reported the effect of marinades of RS extract on beef patties and showed that after 120 s of broiling time, the weight loss was 31%; this value is similar to the results obtained in this study [59]. However, according to Jung and Joo [60], the addition of hibiscus flower did not affect the water retention [60].

### 3.10. Sensory Analysis

Sensory analysis was performed on samples T1, T9, T6, T7 and T8, in order to evaluate the final acceptability of the product by consumers.

**Figure 3:**
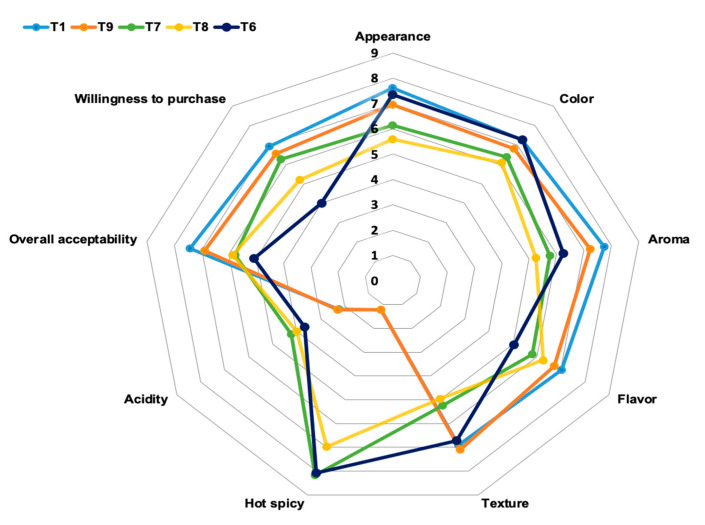
Sensorial results from 40 untrained panelists. Sensorial attributes and purchase intention of beef patties. T1: CM (control), T2: 2% PSW, T3: 4% PSW, T4: 0.35% CA, T5: 2% RS, T6: 2% PSW + 0.35% CA, T7: 2% PSW + 0.35% CA + 2% RS, T8: 4% PSW + 0.35% CA + 2% RS and T9: 0.7% CAMPA N.3 (A).

Figure 3 shows the change of appearance, color, flavor, texture, spicy flavor, acidity, acceptability and purchase intention for the different samples. CM and 0.7% CAMPA N.3 (A) were the most acceptable, thanks to a better aroma and flavor, probably because they were comparable in taste to commercial hamburgers.

The consumer intention to purchase beef patties for T6, T7 and T8 was the lowest; this might be due to the spicy taste due to the addition of CA powder. Appearance and color could also be influenced by the addition of hibiscus flower.

Generally, the CM and 0.7% CAMPA N.3 (A) samples presented the best results. Out of the treatments where a natural powder was added, the highest acceptability and intention to purchase was obtained for T8, possibly because the panelists did not have a habit of eating spicy products.

## 4. Conclusions

The present study proved the benefit of combining PSW, CA and RS in order to prevent lipid oxidation, by achieving the lowest formation of MDA and hexanal in beef patties stored at 4 ± 1 °C, with similar values to the synthetic additive (CAMPA N.3 (A)). Furthermore, the mixes with RS were effective in reducing the microbial activity, % metmyoglobin and incrementing the pH. In addition, the synergy of PSW, CA and RS affected sensory qualities, such as acidity, color, aroma, etc., of the patties.

Based on the above data, PSW, CA and RS represent natural, environmentally friendly and cost-effective alternatives in controlling the oxidative processes and microbial activity in meat products.

Further research is needed to find the conditions to add these kinds of plants in final products without affecting their sensory qualities.

## Figures and Tables

**Table 1:** Formulations for the beef patties.

Treatments
CM (control) (T1)
2% Pecan Shell (PSW) (T2)
4% PSW (T3)
0.35% *Capsicum annuum* (CA) (T4)
2% *Hibiscus sabdariffa* (RS) (T5) 2%PSW + 0.35% CA (T6)
2% PSW + 0.35% CA + 2% RS (T7) 4% PSW + 0.35% CA + 2% RS (T8) 0.7% * CAMPA N.3 (A) (T9)

* CAMPA N.3 (A): (dextrin, dextrose, 5.7% SO_2_, E-224, E-301, E-331).

**Table 2:** Total phenolic content (TPC) and DPPH of PSW, RS and CA extracts.

Sample	TPC (mg GAE/g DW)	DPPH (μmol TE/g DW)
PSW	96.81 ± 0.08 ^a^	104.63 ± 0.01 ^a^
RS	15.02 ±0.01 ^b^	98.29 ± 0.04 ^b^
CA	7.03 ± 0.0 ^c^	73.06 ± 0.01 ^c^

Results are expressed as milligrams of gallic acid equivalent (GAE) per gram of dry sample weight (DW) and micromoles of Trolox equivalent (TE) per gram of DW. Results are expressed as mean ± SD (*n* = 3). Different superscripts in the same column indicate significant differences (*p* < 0.05).

**Table 3:** Antimicrobial activity of PSW, CA, RS (extracts) and controls (+ and −).

Sample	*B. cereus*	*S. aureus*	*L. monocytogenes*	*M. luteus*	*E. coli*	*S. enterica*
CA	−	−	++	++	−	−
PSW	++	++	++	+++	−	++
RS	+++	+++	+++	++++	+++	++
Control + Control −	++ −	+++ −	++++ −	− −	++++ −	++++ −

No antimicrobial activity (−), inhibition zone <1 mm. Slight antimicrobial activity (+), inhibition zone 2–8 mm. Moderate antimicrobial activity (++), inhibition zone 9–15 mm. High antimicrobial activity (+++), inhibition zone 16–22 mm. Strong antimicrobial activity (++++), inhibition zone >23 mm. Control +: water, control−: ampicillin.

**Table 4:** Thiobarbituric acid-reactive substance (TBARS) values (mg MDA/kg) in beef patties during 12 days of refrigerated storage at 4 ± 1 °C.

Day	T1	T2	T3	T4	T5	T6	T7	T8	T9
0	0.27 ± 0.04 ^a^	0.27 ± 0.04 ^a^	0.27 ± 0.04 ^a^	0.27 ± 0.04 ^a^	0.27 ± 0.04 ^a^	0.27 ± 0.04 ^a^	0.27 ± 0.04 ^a^	0.27 ± 0.04 ^a^	0.27 ± 0.04 ^a^
3	0.44 ± 0.07 ^a^	0.198 ± 0.01 ^b^	0.193 ± 0.03 ^b^	0.325 ± 0.02 ^ab^	0.316 ± 0.01 ^ab^	0.201 ± 0.01 ^b^	0.233 ± 0.03 ^b^	0.213 ± 0.01 ^b^	0.241 ± 0.01 ^b^
5	0.69 ± 0.08 ^a^	0.209 ± 0.08 ^bc^	0.140 ± 0.02 ^c^	0.269 ± 0.04 ^bc^	0.382 ± 0.01 ^b^	0.163 ± 0.01 ^bc^	0.285 ± 0.01 ^bc^	0.355 ± 0.02 ^bc^	0.219 ± 0.01 ^bc^
7	0.83 ± 0.07 ^a^	0.195 ± 0.08 ^c^	0.182 ± 0.01 ^a^	0.349 ± 0.01 ^bc^	0.427 ± 0.02 ^b^	0.155 ± 0.02 ^c^	0.306 ± 0.01 ^bc^	0.362 ± 0.03 ^bc^	0.214 ± 0.03 ^bc^
10	1.24 ± 0.07 ^a^	0.313 ± 0.02 ^bcd^	0.223 ± 0.01 ^d^	0.352 ± 0.01 ^bcd^	0.391 ± 0.01 ^bc^	0.213 ± 0.01 ^d^	0.432 ± 0.01 ^b^	0.398 ± 0.02 ^bc^	0.251 ± 0.01 ^cd^
12	1.31 ± 0.07 ^a^	0.454 ± 0.01 ^bcd^	0.434 ± 0.00 ^d^	0.633 ± 0.02 ^bcd^	0.351 ± 0.01 ^bc^	0.573 ± 0.01 ^d^	0.473 ± 0.01 ^b^	0.333 ± 0.01 ^bc^	0.279 ± 0.02 ^cd^

T1: CM (control), T2: 2% PSW, T3: 4% PSW, T4: 0.35% CA, T5: 2% RS, T6: 2%PSW + 0.35% CA, T7: 2% PSW + 0.35% CA + 2% RS, T8: 4% PSW + 0.35% CA + 2% RS and T9: 0.7% CAMPA N.3 (A). Different letters across the same day indicate significant differences between samples. The results are expressed as mg of malondialdehyde (MDA)/kilogram of meat sample.

**Table 5:** Values of hexanal content at day 6 of the experiment.

Sample	Hexanal (μg Hexanal/kg Meat)
CM (T1)	609.3 ± 21.7 ^a^
0.7% CAMPA N.3 (A) (T9)	0.0 ± 0.0 ^b^
2% PSW (T2)	21.4 ± 2.0 ^b^
4% PSW (T3)	43.0 ± 10.0 ^b^
0.35% CA (T4)	4.0 ± 0.0 ^b^
2% RS (T5)	0.0 ± 0.0 ^b^
2% PSW + 0.35% CA + 2% RS (T7)	5.1 ± 0.4 ^b^
4% PSW + 0.35% CA+ 2% RS (T8)	9.2 ± 0.1 ^b^
2% PSW + 0.35% CA (T6)	5.0 ± 0.4 ^b^

Results expressed as mean ± SD (*n* = 3). Different superscripts in the same column indicate samples that differ significantly (*p* < 0.05).

**Table 6:** pH values of the samples at different days of experiment.

Day	T1	T9	T2	T3	T4	T5	T6	T7	T8
0	5.66 ± 0.05 ^cA^	5.71 ± 0.03 ^aA^	5.64 ± 0.05 ^bA^	5.64 ± 0.23 ^bA^	5.70 ± 0.02 ^bA^	5.42 ± 0.04 ^aA^	5.71 ± 0.02 ^bcA^	5.28 ± 0.08 ^aA^	5.20 ±0.10 ^aA^
6	5.87 ± 0.02 ^bA^	5.82 ± 0.06 ^aA^	5.77 ± 0.03 ^bA^	5.79 ± 0.01 ^bA^	5.82 ± 0.01 ^bcA^	5.20 ± 0.05 ^aA^	5.77 ± 0.04 ^bA^	5.27 ± 0.03 ^abA^	5.24 ± 0.01 ^aA^
13	7.54 ± 0.01 ^aA^	5.93 ± 0.07 ^aB^	7.31 ± 0.13 ^aA^	7.33 ± 0.06 ^aA^	7.53 ± 0.08 ^aA^	5.59 ± 0.28 ^aB^	7.45 ± 0.01 ^aA^	5.47 ± 0.01 ^aB^	5.46 ± 0.01 ^aB^

The values are means ± S.D. of the samples analyzed in duplicate. ^a–c^ The means followed by different letters in the same sample, but on different days, indicate significant differences (*p* < 0.05). ^A,B^ The means followed by different letters on the same day, but in different samples, indicate significant differences (*p* < 0.05). T1: CM (control), T2: 2% PSW, T3: 4% PSW, T4: 0.35% CA, T5: 2% RS, T6: 2% PSW + 0.35% CA, T7: 2% PSW + 0.35% CA + 2% RS, T8: 4% PSW + 0.35% CA + 2% RS and T9: 0.7% CAMPA N.3 (A).

**Table 7:** Effect of antioxidants on L*, c* and *h*° parameters of raw beef patties during refrigerated storage (4 ± 1 °C).

	Days	T1	T9	T2	T3	T4	T5	T6	T7	T8
Lightness (L*) A	0	48.32 ± 1.79 ^aA^	50.54 ± 3.30 ^aA^	44.40 ± 3.03 ^aA^	42.16 ± 3.14 ^aA^	50.32 ± 1.68 ^aA^	49.03 ± 1.08 ^aAB^	43.38 ± 4.83 ^aA^	40.87 ± 2.38 ^aA^	38.67 ± 1.82 ^aA^
	6	51.76 ± 3.77 ^aA^	50.03 ± 1.89 ^aA^	44.10 ± 5.3 ^aA^	38.35 ± 2.39 ^aA^	50.55 ± 1.35 ^aA^	53.02 ± 3.26 ^aAB^	49.77 ± 5.67 ^aA^	42.11 ± 5.17 ^aA^	43.12 ± 3.80 ^aA^
	13	44.04 ± 3.83 ^aA^	44.42 ± 5.93 ^aA^	46.95 ± 2.96 ^aA^	41.14 ± 1.37 ^aA^	45.45 ± 1.61 ^aA^	49.53 ± 3.52 ^aAB^	46.65 ± 2.14 ^aA^	41.84 ± 4.73 ^aA^	38.45 ± 1.09 ^bA^
Chroma (c*)	0	47.27 ± 1.23 ^abA^	54.93 ± 4.86 ^aA^	39.73 ± 3.44 ^bA^	27.07 ± 1.20 ^aA^	52.87 ± 0.70 ^aA^	39.30 ± 1.51 ^aA^	35.37 ± 2.70 ^aA^	32.37 ± 4.12 ^aA^	26.03 ± 2.04 ^aA^
	6	31.14 ± 5.19 ^aCD^	45.02 ± 7.50 ^aABC^	24.63 ± 2.29 ^bBC^	11.22 ± 0.61 ^aBC^	32.77 ± 2.26 ^bDE^	19.31 ± 3.28 ^aCD^	16.29 ± 3.13 ^bBCD^	19.41 ± 2.71 ^aBC^	11.20 ± 0.97 ^bBC^
	13	33.86 ± 1.98 ^aBCD^	34.68 ± 6.08 ^aC^	17.47 ± 5.96 ^abC^	9.62 ± 4.14 ^bC^	34.22 ± 3.19 ^abCDE^	11.18 ± 4.41 ^bD^	9.16 ± 1.32 ^cE^	16.19 ± 2.63 ^bC^	7.55 ± 0.54 ^cC^
Hue angle (*h*°)	0	19.02 ± 3.87 ^aBC^	25.17 ± 4.60 ^aABC^	19.03 ± 0.38 ^abBCD^	11.94 ± 7.21 ^aA^	24.16 ± 2.17 ^aABC^	24.89 ± 2.23 ^aAB^	17.69 ± 1.41 ^aAB^	16.51 ± 2.80 ^aDE^	17.17 ± 6.20 ^abA^
	6	33.02 ± 7.22 ^aABC^	29.99 ± 1.22 ^aAB^	32.58 ± 3.79 ^aABC^	25.19 ± 6.11 ^aA^	38.02 ± 4.71 ^aA^	45.51 ± 6.06 ^aA^	38.79 ± 4.12 ^aA^	37.60 ± 5.98 ^aAB^	28.90 ± 2.57 ^aA^
	13	24.09 ± 6.85 ^aABC^	26.11 ± 2.13 ^aABC^	25.03 ± 6.10 ^aABCD^	11.21 ± 2.11 ^aA^	20.97 ± 6.23 ^aBC^	38.54 ± 2.30 ^aAB^	8.24 ± 2.10 ^aB^	32.86 ± 3.74 ^aABC^	24.29 ± 6.77 ^aC^

The values are means ± S.D. of the samples analyzed in duplicate. ^a,b,c^ The means followed by different letters in the same sample but on different days indicate significant differences (*p* < 0.05). ^A,B,C^ The means followed by different letters on the same day but in different samples indicate significant differences (*p* < 0.05). T1: CM (control), T2: 2% PSW, T3: 4% PSW, T4: 0.35% CA, T5: 2% RS, T6: 2% PSW + 0.35% CA, T7: 2% PSW + 0.35% CA + 2% RS, T8: 4% PSW + 0.35% CA + 2% RS and T9: 0.7% CAMPA N.3 (A).

**Table 8:** Cooking loss (%) of beef patty cooking in different treatments.

Sample	Cooking Loss (%)
T1	26.10 ± 1.22 ^a^
T6	28.03 ± 1.18 ^a^
T7	28.30 ± 0.48 ^a^
T8	27.95 ± 1.38 ^a^
T9	28.10 ± 0.71 ^a^

The values are means ± S.D. of the samples analyzed. ^a^ The means no significant differences (*p* < 0.05) of cooking loss (%) between samples. T1: CM (control), T6: 2% PSW + 0.35% CA, T7: 2% PSW + 0.35% CA + 2% RS, T8: 4% PSW + 0.35% CA + 2% RS and T9: 0.7% CAMPA N.3 (A).

## Abbreviations

PSW	Pecan shell (*Carya illinoinensis*)
RS	Roselle flower (*Hibiscus sabdariffa*)
CA	Red pepper (*Capsicum annuum*)
TE	Trolox equivalent
DW	Dry weight
GAE	Gallic acid equivalent
TPC	Total phenolic compounds
CM	Control
CFU	Colony-forming unit
TBARS	Thiobarbituric acid-reactive substances
MDA	Malondialdehyde
BHA	Butilate hidroxianilose
BHT	Butilate hidroxitoluene
PUFA	Polyunsaturated fatty acids
MUFA	Monounsaturated fatty acids
SFA	Saturated fatty acids
